# Immune Cells, Superheroes or Villains? Targeting the Bad Guys to Treat Myxomatous Mitral Valve Disease∗

**DOI:** 10.1016/j.jacbts.2022.06.020

**Published:** 2022-11-14

**Authors:** Joy Lincoln

**Affiliations:** Department of Pediatrics, Section of Pediatric Cardiology, Medical College of Wisconsin, Milwaukee, Wisconsin, USA; The Herma Heart Institute, Children’s Wisconsin, Milwaukee, Wisconsin, USA

**Keywords:** CCR2, extracellular matrix, inflammation, myxomatous valve disease

**Figure:**
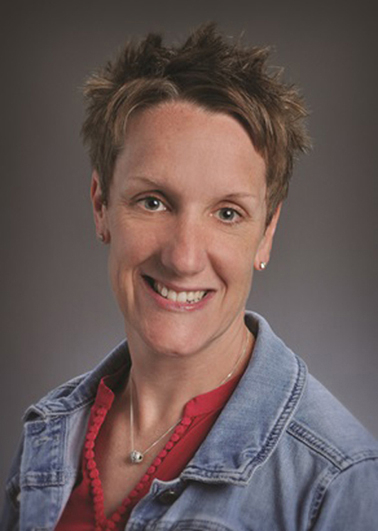

Heart valves open and close over 100,000 times a day over the course of ∼80 years in the average healthy human to ensure unidirectional blood flow. This critical function is dependent upon the preservation of a highly organized extracellular matrix (ECM) network within the valve leaflets/cusps and supporting apparatus. This includes the discrete assembly and localization of collagen fibers, proteoglycans, and elastin fibers that provide strength, compressibility, and extensibility, respectively, in response to dynamic changes in the biomechanical environment with every cardiac cycle. As an accepted generalization, aberrations in the abundance, organization, and quality of the ECM components results in instability of the valve infrastructure, leading to gross functional impairment (reviewed by Kodigepalli et al^1^). Nowhere is this more prominent than in patients with heritable connective tissue disorders caused by alterations in ECM genes. An example of this is Marfan syndrome, an autosomal dominant condition affecting 1 in 3,000 to 5,000 individuals presented with a broad range of clinical manifestations, including myxomatous mitral valve disease. Studies have reported that approximately 40% to 50% of those with Marfan syndrome develop myxomatous valve degeneration characterized by elastin and collagen fiber disruption and an exuberant accumulation of proteoglycans within left-sided valve leaflets. As a result, affected leaflets become thickened and mechanically incompetent as determined by leaflet billowing, malcoaptation, and chordal rupture, referred to as mitral valve prolapse. Furthermore, secondary complications include left ventricular remodeling and dysfunction, heart failure, arrhythmogenesis, and sudden cardiac death if left untreated. At present, mitral valve repair or replacement is the most effective treatment; yet, this comes with insuperable complications particularly in aging or predisposed patient populations. Therefore, for many decades, the field has searched for better therapeutic alternatives beyond surgical repair to improve patient outcomes and quality of life.

In parallel with the development of improved mitral valve surgery therapies, the field is eager to advance the application of mechanistic-based approaches to prevent and treat the early myxomatous changes that underlie the progression to functional prolapse. However, delineating the cellular and molecular underpinnings of the myxomatous process within the valve structures in human patients is a challenge, and as a result, therapeutic advancements beyond surgery have been hindered. However, the development of mouse models that recapitulate human phenotypes seen in some of the more common connective tissue disorders in humans have provided investigators with powerful tools to investigate mechanistic insights of syndromic valve disease pathogenesis. In 2004, the laboratory of Dr Hal Dietz generated a mouse model of Marfan syndrome based on the human equivalent of a genetic mutation in the *Fibrillin-1* (*FBN1*) gene.^2^ At a young age, mice homozygous for the cysteine to glycine substitution at nucleotide 1039 develop mitral valve thickening by postnatal day 4.5 as well as aortic disease, including root dilation, aneurysm development, and dissection, similar to humans, although in mice, dissection evokes premature lethality around postnatal day 7. However, heterozygous (*Fbn1*^*C1039/+*^) animals are viable, and Ng et al^2^ reported mitral valve thickening by postnatal day 6.5 with histological characteristics of a myxomatous valve phenotype and associated prolapse ∼9 months. In 2020, the same authors of this editorial focus^3^ used Dietz’s *Fbn1*^*C1039/+*^ mouse model to further characterize the pathobiology of myxomatous valve disease, and additionally included gene-edited heterozygous Marfan syndrome pigs (*Fbn1*^*Glu433AsnfsX98/+*^). The study intriguingly reported the presence of a novel proinflammatory environment within regions of myxomatous ECM changes as evident by expansion of tissue resident macrophages, and infiltration of bone marrow-derived monocytes and macrophages. Furthermore, this 2020 study advanced the field by demonstrating that genetic depletion of the monocyte-specific C-C chemokine receptor type 2 (*Ccr2*^*RFP/RFP*^) in the *Fbn1*^*C1039/+*^ background was protective against the reported myxomatous mitral valve phenotypes, but interestingly not aneurysm formation. Although innovative observations were made from this study, the translational value of genetically targeting the permanent inactivation of CCR2 in all cells throughout life (from embryo to adult) in Marfan syndrome patients could be limiting.

In this issue of *JACC*: *Basic to Translational Science*, Xu and Yutzey^4^ dig deeper into the role of immune cells in mitral valve disease by mechanistically demonstrating that immune processes are the main pathogenic drivers of mitral valve disease in the *Fbn1^C1039/fl^* mouse model of Marfan syndrome. The authors applied bulk RNA-seq and showed that the phenotypic “rescue” of mitral valve disease in the previously reported CCR2-deficient *Fbn1*^*C1039/+*^ mice^3^ is caused by restored low levels of inflammatory-enriched mRNAs, including associated inflammatory proteases (chymases, carboxypeptidases). Specifically, amelioration of the inflammatory response in CCR2-deficient *Fbn1*^*C1039/+*^ mice was found to localize to the site of abnormal proteoglycan accumulation around the tip of the leaflet. These experimental outcomes alone provide novel and significant insights into the key processes that underlie mitral valve disease, not only in the Marfan population, but across the spectrum of valve diseases associated with pathological ECM remodeling or myxomatous changes. These transformative findings demonstrating that CCR2+ immune cells are required for pathological ECM remodeling within the degenerating mitral valve create the foundation for future in-depth studies investigating how these cells physically disrupt ECM homeostasis, although here, the authors reasonably speculate that immune cell-dependent proteolytic functions play a role.

In addition to providing a mechanistic understanding of myxomatous changes in diseased valves, the current study overcomes previous translational limitations of genetic approaches to ablate CCR2 by treating affected *Fbn1*^*C1039/+*^ mice with the pharmacological antagonist RS504393. Administration of the drug to 1-month-old *Fbn1*^*C1039/+*^ mice (before CCR2+ increases) for 30 days substantially prevented infiltration of CCR2+ immune cells as well as CD45+ leukocytes and MHCII macrophages. In association with a “dampened” immune response, the increase in inflammatory proteases and myxomatous ECM changes observed in mitral valves from *Fbn1*^*C1039/+*^ mice was not seen following RS504393 treatment. These preclinical animal studies pave the way for developing preventative therapeutic protocols in Marfan syndrome patients. Although prevention is imperative, the penetrance of mitral valve prolapse in Marfan patients is not 100% (40%-50%), and as the risk stratification is not well defined, it is difficult to predict which individual patients will go on to develop significant valve dysfunction to the point of requiring surgical intervention. Notably, we have learned from studies in *Fbn1*^*C1039/+*^ mice that intrinsic deterioration of the valve structure at 2 months of age^3^ precedes functional impairment observed from 9 months.^2^ Therefore, echocardiographic analysis alone may not be sufficient to identify those at-risk patients early, before the development of valvular prolapse and debilitating secondary complications. However here, Xu and Yutzey^4^ also reported the clinical potential of implementing RS504393 as a treatment strategy for myxomatous valve degeneration by injecting 6-month-old *Fbn1*^*C1039/+*^ mice for 30 days, which successfully restored myxomatous changes including leaflet thickness and excess proteoglycan accumulation.

Thus, for the first time, the field has identified a novel target (CCR2) for therapeutic intervention in the treatment of myxomatous valve disease, at least in the setting of Marfan syndrome, and further used an existing preclinical,^5^ selective CCR2 antagonist to prevent and treat valvular disease in a relevant mouse model of human pathology. From here, we are gifted with the knowledge of applying what has been learned about the contribution of immune processes in disrupting ECM homeostasis, and we await the clinical application of RS504393 and other immune-based cytokine inhibitors in the treatment of other hereditary and acquired valve pathologies.

## Funding Support and Author Disclosures

The author has reported that she has no relationships relevant to the contents of this paper to disclose.
